# A regressive machine-learning approach to the non-linear complex FAST model for hybrid floating offshore wind turbines with integrated oscillating water columns

**DOI:** 10.1038/s41598-023-28703-z

**Published:** 2023-01-27

**Authors:** Irfan Ahmad, Fares M’zoughi, Payam Aboutalebi, Izaskun Garrido, Aitor J. Garrido

**Affiliations:** Automatic Control Group-ACG, Institute of Research and Development of Processes-IIDP, Department of Automatic Control and Systems Engineering, Faculty of Engineering of Bilbao EIB/BIE, The University of the Basque Country-UPV/EHU, Po Rafael Moreno no3, Bilbao, 48013 Spain

**Keywords:** Ocean sciences, Energy science and technology, Power stations, Engineering, Electrical and electronic engineering, Energy infrastructure

## Abstract

Offshore wind energy is getting increasing attention as a clean alternative to the currently scarce fossil fuels mainly used in Europe’s electricity supply. The further development and implementation of this kind of technology will help fighting global warming, allowing a more sustainable and decarbonized power generation. In this sense, the integration of Floating Offshore Wind Turbines (FOWTs) with Oscillating Water Columns (OWCs) devices arise as a promising solution for hybrid renewable energy production. In these systems, OWC modules are employed not only for wave energy generation but also for FOWTs stabilization and cost-efficiency. Nevertheless, analyzing and understanding the aero-hydro-servo-elastic floating structure control performance composes an intricate and challenging task. Even more, given the dynamical complexity increase that involves the incorporation of OWCs within the FOWT platform. In this regard, although some time and frequency domain models have been developed, they are complex, computationally inefficient and not suitable for neither real-time nor feedback control. In this context, this work presents a novel control-oriented regressive model for hybrid FOWT-OWCs platforms. The main objective is to take advantage of the predictive and forecasting capabilities of the deep-layered artificial neural networks (ANNs), jointly with their computational simplicity, to develop a feasible control-oriented and lightweight model compared to the aforementioned complex dynamical models. In order to achieve this objective, a deep-layered ANN model has been designed and trained to match the hybrid platform’s structural performance. Then, the obtained scheme has been benchmarked against standard Multisurf-Wamit-FAST 5MW FOWT output data for different challenging scenarios in order to validate the model. The results demonstrate the adequate performance and accuracy of the proposed ANN control-oriented model, providing a great alternative for complex non-linear models traditionally used and allowing the implementation of advanced control schemes in a computationally convenient, straightforward, and easy way.

## Introduction

**Figure 1:**
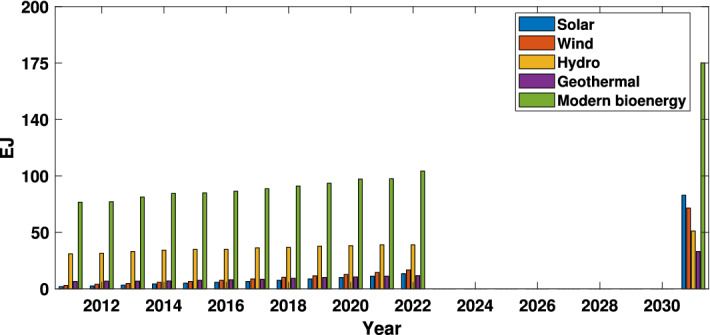
Renewable electricity generation growth by technology net scenario, 2010–2030.

As a result of climate change, emerging markets and developing economies’ energy consumption increased by 4.6% in 2021, according to the Global Energy Research^1^. Therefore, the world is rushing toward clean energy resources to cope with energy demands, as illustrated in Fig. 1. Wind and wave power are two of the most important renewable energy sources for the power industry. According to the European Marine System strategic road-map, the ocean energy infrastructure for Europe will meet almost 10% of Europe’s power consumption from wind, wave and tidal energy by 2050^2^. Therefore, several countries, including the United Kingdom and Spain, have been involved in various projects based on the development of wind energy^3^ and Wave Energy Converters (WECs)^4^. Offshore wind turbines provide the best alternative, with an edge of higher wind quality than onshore. As a result, offshore electricity production has remarkably increased^5,6^.

**Figure 2:**
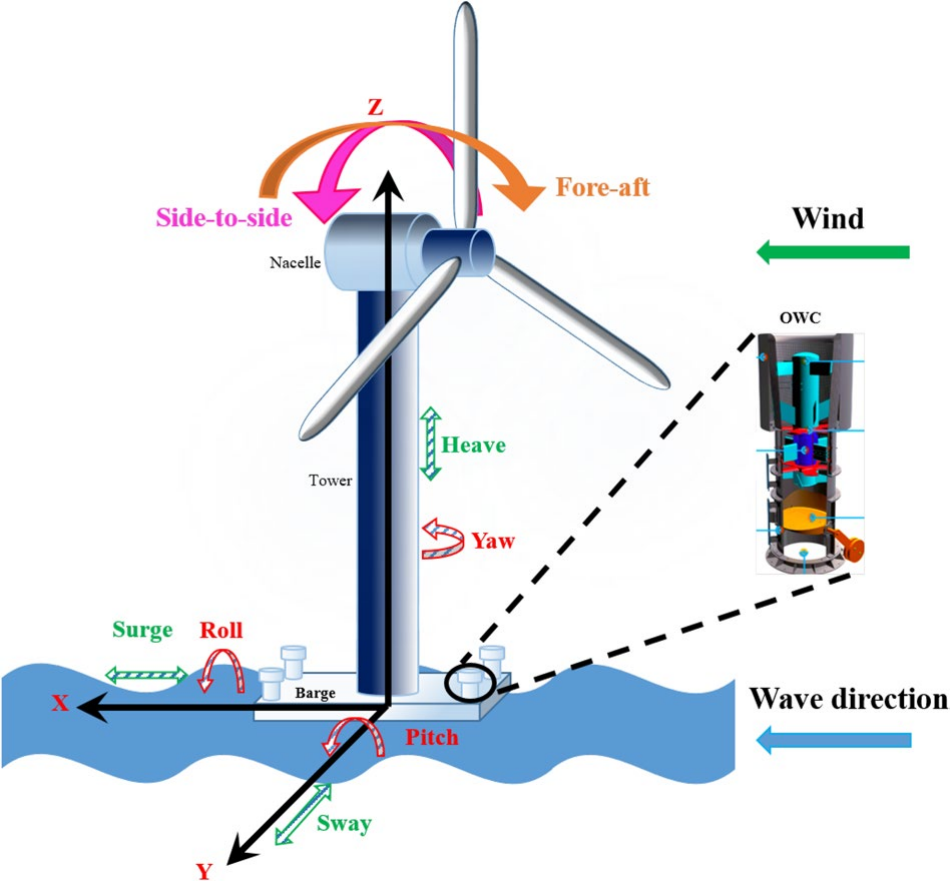
Barge-based floating offshore wind turbine with four OWCs.

FOWTs work on the principle of the law of conservation of energy by converting mechanical energy into electrical energy that is then used to spin electrical generators to produce electrical power. Offshore wind speeds are often higher, and even a slight rate increase can result in significant growth in energy generation^7^. Besides, a specific type of WEC called an OWC can be integrated into the FOWT structure. The operating principle of the OWC consists of an enclosed chamber with an opening beneath, which allows water to flow upwards and downwards, according to the incoming waves. In this way, the air inside the chamber is compressed and decompressed, propelling self-rectifying air turbines located in the upper part of the chambers. This same technology is currently being used in Mutriku’s MOWC wave power plant^8,9^. Therefore, FOWTs and OWCs compose some of the most promising technologies for harnessing clean energy and could be combined in a hybrid platform, as shown in Fig. 2.

Nevertheless, the stresses and fatigue induced by winds and waves have a negative impact on the lifespan of structures of floating platforms^10^. These undesired vibrations cause efficiency reduction, structural imbalance, high maintenance costs, and, eventually, lead to equipment failures^11^. Numerous researchers are working on this potential area to mitigate the aforementioned undesired motions^12,13^. Several researchers from various domains are working to enhance the overall performance of floating structures. Examples include forecasting/prediction of model states, design optimization, fault diagnostics, and developing optimal control of wind turbines. Hybrid platform stabilization is a challenging task and a variety of approaches are employed to mitigate the undesired vibrations of platforms. Passive or active structural control is considered the most convenient way to reduce the load on floating wind turbines. The recently published articles present techniques for wind turbine stabilization using closed-loop control on the hybrid platform^12,14^. Some methods for barge platform stabilization are Tuned Mass Dampers (TMD)^15^, inerters^16^, Liquid Mass Dampers (LMD)^17^ and through mooring lines^18^. For example, Jonkman et al.^19,20^ used a gain-scheduled proportional-integral technique in the development of FAST (Fatigue, Aerodynamics, Structures, and Turbulence) simulator and designed a baseline collective blade pitch controller for three primary floating wind turbines. The detailed characteristics of the floating offshore wind turbine considered in this manuscript, are presented in Table 1.

Hybrid systems are equipped with a range of tools to measure vibration, temperature, humidity, and other variables. These data collection systems measure every parameter to assess the state of the system. Then robust algorithms extract as much information as possible from the available data. A significant amount of data may be processed by machine learning algorithms, with ANNs being one of the most popular techniques modelings of such nonlinear systems^21^. There have been several regression-oriented approaches with various training functions used^22,23^. The Levenberg-Marquardt backpropagation^24^ and Bayesian regularization backpropagation^25^ algorithms are regarded as the best options for such nonlinear dynamics due to their fast computation because fast backpropagation algorithms are highly recommended as first-choice supervised algorithms. It has been shown that ANNs are effective when physical processes are obscure or complicated^26^. As a result, several researchers used ANN-oriented algorithms to improve the overall performance of floating structures. Adaptive learning, self-organization, fault tolerance, online operation, and ease of system integration are a few benefits of adopting ANNs. For example, Multilayer Perceptron (MLP)-based technique for forecasting wind speed at various locations inside a wind farm was developed by Salcedo-Sanz et al.^27^. They demonstrated that this approach yields minimal mean absolute error values in a real wind farm. Several studies on short-term wind speed forecasting use various models, such as two-layers ANN^28^, IRBFNN^29^, RBFNN^30^, non-linear adaptive model^31^, an ensemble of mixture density ANN networks^32^, deep ANN^33^, adaptive boosting (adaboosting) ANN^11^, etc. These studies show that most ANN-based models are more accurate than methods that do not use artificial intelligence. The best model for each case is determined by the type of data and the estimation criteria. There are numerous forecasting studies for wind power in the short, medium, and long term in the literature. Ma et al. proposed a hybrid method involving a generalized dynamic fuzzy neural network^34^.

Dong et al.^35^ suggested a hybrid model combining an integrated processing strategy and a linear neuro-fuzzy function to forecast wind power. The accuracy of the method presents results that are 5.33% more accurate than results obtained with ANN. Other hybrid models are based on neuro-fuzzy^36^, GA-BP NN^37^, wavelet ANN^38^ and Adaptive Wavelet ANN^39^. Short-term wind power prediction methods are based on BPNN^40^, convolutional and recurrent ANNs^41^, Elman ANN^42^, Boltzmann machine^43^ and artificial bee colony ANN^44^. However, no model has so far been developed for FOWT coupled to OWCs that are suitable for closed-loop control. Recently, M’zoughi et al.^45^ demonstrated the feasibility of integrating two OWCs and Aboutalebi et al.^46^ the feasibility of four OWCs in barge platforms. Integrating four OWCs into a platform arises as a promising solution as an active structure control (Fig. 2)^46^. The size and geometry of the barge platform make it more favorable to create space for wave energy converter integration compared to spar and tension leg platforms.

The main significant novelty in this work relies on the use of a control-oriented artificial neural networks model for the hybrid wave and wind barge platform. To do so, an efficient, intelligent machine learning modelling is required so as to assess the effectiveness of novel platform designs and replicate the complex hybrid dynamics of FOWT-OWCs, which will allow the use of advanced feedback controllers. Hence, an intelligent control-oriented model has been developed for the hybrid platform that was also developed from scratch since, in contrast with simple FOWT systems, there were not dynamical models within FAST for this kind of systems. Even, when some efforts have been deployed into hybrid platforms for energy generation related to semi-submersible platforms in the time or frequency domain^47–49^, there is no research in control of hybrid FOWT-OWCs for platform stabilization. Therefore, the main novelty of the work relays in the development of an intelligent control-oriented model for a new hybrid system, suitable to mitigate undesired vibrations from the platform by means of closed-loop control schemes. That is, using the oscillating water columns to implement platform stabilization with active structural controllers.

The rest of the manuscript has been organized as follows: The theoretical and mathematical concepts have been summarized in section “Theoretical background”. Section “Methods” explains the process of four OWCs geometry designs and advanced computations with WAMIT and FAST software. In section “ANN-based FOWT model”, the ANN-based FOWT model have been developed. Section “Computations and results” presents the simulations and results, including the corresponding validation. Finally, the last section presents the conclusions.

**Table 1:** 5-MW FOWT features.

Parameter	Value
Hub height	90 m
Center of mass location	38.23 m
Rotor diameter	126 m
Number of blades	3
Initial rotational speed	12.1 rpm
Blades mass	53.22 kg
Nacelle mass	240,000 kg
Hub mass	56,780 kg
Tower mass	347,460 kg
Power output	5 MW
Cut-in, Rated, Cut-out wind speed	3 m/s, 11.4 m/s, 25 m/s

## Theoretical background

The input to the FOWT model is considered as unidirectional regular waves and can be represented as^50^ (1):

1 $$\begin{aligned} g(t) = Asin (\omega t) = A\sin (2\pi ft) = A\sin \left( {\frac{{2\pi }}{\lambda }ct} \right) \end{aligned}$$

where the propagation speed is $c=\lambda f$. $\lambda$ is the wavelength, which is the distance between successive crests, and *A* is the wave amplitude from Still Water Level (*SWL*) to the wave crest. To improve the accuracy of the coupled simulation by including a nonlinear irregular wave model that is more appropriate for shallow water depths, where most offshore wind turbines are sited. Therefore, the nonlinear irregular wave model is incorporated in the coupled aero-servo-hydro-elastic simulation of a hybrid FOWT-OWCs system. The two most widely recognized theoretical wave spectra are the Bretschneider spectrum for fully developed waves and the JONSWAP spectrum for partially developed waves. The generalized representation of the spectrum can be expressed as^51^.

2 $$\begin{aligned} S_i (\omega _i ) = (1 - 0.287\ln (\gamma ))\frac{{5\omega _p^4 }}{{16\omega ^5 }}K_s^2 \gamma ^\beta e^{\frac{{5\omega _p^4 }}{{16\omega ^5 }}} \end{aligned}$$

where $\beta$ is $\exp ( - \frac{{(\omega - \omega _p )^2 }}{{2\omega _P^2 \alpha ^2 }})$, $\alpha$ is $$\left\{ \begin{array}{l} 0.07,\,\,\,\,\,\,if\,\omega \le \omega _p \\ 0.09,\,\,\,\,\,\,if\,\omega \le \omega _p \\ \end{array} \right\}$$, $K_s$ is the wave height, and $\omega _p$ is the peak angular frequency. Depending on the wave condition, a value between 1 and 5 is chosen for the heat ratio parameter. The value of the standard JONSWAP spectrum $\gamma$ is 3.3^52^.

The nonlinear dynamics of a 5-MW FOWT integrated with four OWCs to barge a platform in the time-domain can be described as (3):

3 $$\begin{aligned} M_{ij} (x,u,t)\ddot{x}_j = f_i (x,\dot{x},u,t) \end{aligned}$$

where $M_{ij}$, is the mass inertia, *t* is the time, *u* is the control inputs, and $\ddot{x}$ is the second time derivative of the $j\mathrm{th}$ Degree of Freedom (DOF).

The generalized outside force acting on the system is represented by the term on the right-hand side of Eq. (3), which includes the aerodynamic load on the blades and nacelle, hydrodynamic forces on the platform, elastic and servo forces. In the frequency domain, the generalized system for the linear equations of motion can be expressed as:

4 $$\begin{aligned} I_{FOWT} (\omega )\ddot{x} + D_{FOWT} (\omega )\dot{x} + S_{FOWT} x = \vec f_{FOWT} (\omega ) + \vec f_{PTO} (\omega )\ \end{aligned}$$

where $I_{FOWT}$, $D_{FOWT}$, and $S_{FOWT}$ may be represented as inertia, damping, and stiffness matrices, respectively. $\vec f_{PTO} (\omega )$ and $\vec f_{FOWT} (\omega )$ represented as the drag of waves and hydrodynamic forces imposed by Power-take-off (PTO). A column vector of nonlinear state-space can be deduced from Eq. (4) may be represented as:

5 $$\begin{aligned} \ x = \left[ {\begin{array}{*{20}l} {roll} \\ {pitch} \\ {yaw} \\ {surge} \\ {sway} \\ {heave} \\ {fore\text {-}aft} \\ {side\text {-}to\text {-}side} \\ \end{array}} \right] \end{aligned}$$

The inertia, damping and stiffness matrices of the FOWT can be best expressed in the following Eqs. (6–8).

6 $$\begin{aligned}{}&I_{FOWT} (\omega ) = A_{Hydro} (\omega ) + M_{Platform} + M_{Tower} \end{aligned}$$

7 $$\begin{aligned}{}&D_{FOWT}(\omega )=D_{Hydro}(\omega )+D_{Tower}+D_{viscous}+D_{chamber} \end{aligned}$$

8 $$\begin{aligned}{}&S_{FOWT} = S_{Hydro} + S_{Mooring} + S_{Tower} \end{aligned}$$

The coefficient of inertia, $I_{FOWT} (\omega )$ for FOWT, $A_{Hydro} (\omega )$ represents the platform’s added mass, $M_{Platform}$ and $M_{Tower}$ are the platform and tower mass matrices, respectively. In the damping matrix $D_{FOWT}(\omega )$ for FOWT, $D_{Hydro}(\omega )$, $D_{Tower}$, and $D_{viscous}$ denote the floating platform damping, flexible tower matrix, and viscous drag, respectively. $D_{chamber}$ indicates the external damping caused by PTO’s effect on overall dynamics. The stiffness matrices $S_{FOWT}$, $S_{Hydro}$, $S_{Mooring}$ and $S_{Tower}$ are defined as, the platform’s hydrostatic restoring matrix, mooring lines, spring stiffness and the tower stiffness coefficients matrix, respectively.

For the four integrated OWCs, the pressure inside the chamber is uniform when assuming that the internal free surface behaves like a piston. As a result, the external force is defined as follows:

9 $$\begin{aligned} f_{PTO}(\omega )=- \nu (\omega )S \end{aligned}$$

where $\nu$ is the pressure drop across the turbine and *S* is the internal free surface area. The relationship between air density and pressure is an isentropic transformation.

10 $$\begin{aligned} \rho = \rho _a \left( {\frac{\nu }{{\nu _a }}} \right) ^{\frac{1}{\gamma }} \end{aligned}$$

where $\rho _a$ and ${\nu _a}$ are the density and pressure of the chamber in its resting condition, and $\gamma$ is the heat capacity ratio of air. The following equation is obtained from the derivative of the linearized form of Eq. (10):

11 $$\begin{aligned} \dot{\rho }= \frac{{\rho _a }}{{\gamma \nu _a }}\dot{\nu } \end{aligned}$$

The linearized mass flow inside the turbine can be calculated as:

12 $$\begin{aligned} \dot{m} = \frac{{d(\rho V)}}{{dt}} = \frac{{\rho _a }}{{\gamma p_a }}\dot{p}V_a + \rho _a \dot{V} \end{aligned}$$

where $V_a$ is the air volume in the chamber in an undisturbed state and *V* is the air volume variation in the chamber. A Wells turbine with a diameter of *D* and a rotational velocity of *N* is described by a linear relationship between the pressure and flow coefficients.

13 $$\begin{aligned} \psi = \mathrm{K}\zeta \end{aligned}$$

where the pressure and flow coefficients are given as:

14 $$\begin{aligned} \psi= & {} \frac{\nu }{{\rho _a N^2 D^2 }} \end{aligned}$$

15 $$\begin{aligned} \zeta= & {} \frac{{\dot{m}}}{{\rho _a ND^3 }} \end{aligned}$$

The flow rate determines the pressure drop. As a result of the non-dimensionalization, the linear relationship is defined as follows:

16 $$\begin{aligned} \psi _c = \mathrm{K}_c\zeta _c \end{aligned}$$

where the pressure and flow coefficients can be expressed as:

17 $$\begin{aligned} \psi _c=\frac{\nu }{{\rho _a g H}} \end{aligned}$$

18 $$\begin{aligned} \zeta _c=\frac{{2\pi \dot{m}}}{{\rho _a \omega S H}} \end{aligned}$$

and *g* represents the gravitational acceleration value. As a result of incorporating Eqs. (13–15) into Eq. (12), the mass flow inside the turbine is described as follows:

19 $$\begin{aligned} \dot{m}(\omega ) = \frac{{S\omega }}{{2\pi gK_c }} \end{aligned}$$

The pressure complex amplitude can be expressed by combining Eqs. (12) and (19).

20 $$\begin{aligned} \hat{\nu }(\omega ) = i\omega \frac{\Upsilon }{{S\omega \left[ {1 + \left( {\varepsilon \Upsilon } \right) ^2 } \right] }}\hat{V} - \omega ^2 \frac{{\varepsilon \Upsilon ^2 }}{{S\omega \left[ {1 + \left( {\varepsilon \Upsilon } \right) ^2 } \right] }}\hat{V} \end{aligned}$$

where $\hat{V}$ is the complex amplitude of the air volume oscillation and the constants $\Upsilon$ and $\varepsilon$ are given by:

21 $$\begin{aligned} \Upsilon= & {} 2\pi \rho _a gK_c \end{aligned}$$

22 $$\begin{aligned} \varepsilon= & {} \frac{{V_a }}{{\Upsilon \nu _a S}} \end{aligned}$$

According to Eqs. (12) and (19), the PTO force is described as:

23 $$\begin{aligned} \hat{f}_{PTO}(\omega ) = - i\omega B_{PTO} \hat{x}_r + \omega ^2 K_{PTO} \hat{x}_r \end{aligned}$$

where $\hat{x}_r$ is the complex amplitude of the relative displacement. According to the aforementioned Eq. (23), the PTO damping and stiffness coefficients are as follows^53^:

24 $$\begin{aligned} B_{PTO}(\omega ) = \frac{{\Upsilon S}}{{\omega \left[ {1 + \left( {\varepsilon \Upsilon } \right) ^2 } \right] }} \end{aligned}$$

and

25 $$\begin{aligned} K_{PTO}(\omega ) = \frac{{\varepsilon \Upsilon ^2 S}}{{\omega ^2 \left[ {1 + \left( {\varepsilon \Upsilon } \right) ^2 } \right] }} \end{aligned}$$

Eventually, in the frequency domain, the system of motion equations for the FOWT, provided by Eq. (4), may be represented as:

26 $$\begin{aligned} I_{FOWT} (\omega ) \mathop {\hat{x}}\limits ^{ \cdot \cdot } + (D_{FOWT} (\omega )+B_{PTO} (\omega ))\mathop {\hat{x}}\limits ^ \cdot + (S_{FOWT} +K_{PTO} (\omega ))\hat{x} = \vec f_{FOWT} (\omega ) \end{aligned}$$

Equation (26) has a term on the right-hand side that is defined as:

27 $$\begin{aligned} \vec f_{FOWT} (\omega ) = \vec f_{Hydro} (\omega ) + \vec f_{viscous} (\omega ) \end{aligned}$$

where the viscous force is $\vec f_{viscous}$, while the hydrodynamic force of the waves on the platform is $\vec f_{Hydro}$.

According to a theoretical study, an OWC device’s maximum primary conversion efficiency can reach 70% under ideal damping conditions (Evans and Porter^54^]). However, in practice, the wave energy conversion efficiency of an actual wave energy converter is typically lower (or substantially lower). Relevant wave energy conversion efficiencies for a number of OWC devices evaluated in wave tanks/flumes have been included in Table 2, showing significant variations from one another. In this regard,^55^ claims that a generic OWC has a relatively poor wave energy conversion efficiency of 7.5%, while in studies^56^ the efficiency was found to be 35%. Wells turbine is limited to avoid stalling behaviour. In these study^14,57^, researchers introduced stalling-free techniques of power extraction applied to the case of the Nereida project plant, located on the Basque coast of Mutriku.

**Table 2:** Primary conversion efficiency for different OWCs.

	OWC type	AC area^b^ (m^2^)	$\eta _{max}$ (%)	Period *T*(*s*)	References
1	Floating	0.166	35	1.67	^56^
2	BBDB^a^	0.156	65	1.48	^58^
3	Fixed	0.96	70	1.69	^59^
4	Cylinder	0.0085	7.5	1.25	^55^

^a^ Backward Bent Duct Buoy

^b^Sectional area of the air chamber

The hydrodynamic performance of the OWC device is evaluated by analyzing the hydrodynamic efficiency. The power available at the turbine inlet during the period of a wave cycle is provided by

28 $$\begin{aligned} P_t = \frac{1}{T}\int \limits _t^{t + T} {\int \limits _s {P_c (t)v_i(t)dsdt} } \end{aligned}$$

where $v_i(t)$ and $P_c$ are the instantaneous velocity and instantaneous value of chamber pressure, respectively. The power of the incident wave is defined as^60^:

29 $$\begin{aligned} E = \int \limits _{ - h}^q {P_D \cdot udy} \end{aligned}$$

here $P_D$ is the dynamic pressure, *h* is the water depth below the SWL and *q* is the free surface elevation. By averaging over a wave period, average energy flux $P_E$ can be calculated as, which is represented by 

30 $$\begin{aligned} P_E = \frac{1}{{}}\int \limits _t^{t + T} {\int \limits _{ - h}^q {P_D .udy} dt = } \frac{1}{T}\int \limits _t^{t + T} {\int \limits _{ - h}^q {(P + \rho gy)udy} dt} \end{aligned}$$

where *g* is the gravitational acceleration. The hydrodynamic efficiency of the OWC device is given by

31 $$\begin{aligned} \eta _{owc} = \frac{{P_t }}{{P_E }} \end{aligned}$$

## Methods

The barge platform developed by Jonkman et al.^19^ is a standard 40 m $\times$ 40 m $\times$ 10 m with a closed moonpool at the center. Therefore, it is necessary to integrate the four moonpools of OWCs for platform stabilization using computational numerical tools i.e. MultiSurf and WAMIT, in order to couple the resulting surface to FAST.

**Figure 3:**
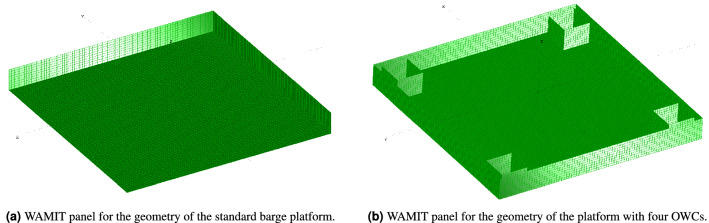
Platforms geometry design.

### Geometry design

The geometry of the proposed platform is designed using MultiSurf. Two different platforms with distinct features have been evaluated. The first platform, as depicted in Fig. 3a, is a regular barge platform, whereas the second platform, as shown in Fig. 3b, is a barge platform with four OWCs in the corners. The standard platform is constructed with 2240 rectangular panels within a quarter of the body. On the other hand, four moonpools have been incorporated and the platform modeled with 2240 rectangular panels according to their coordinates. Each OWC is installed at a distance of 1 m from the sides with the dimensions 5 m $\times$ 5 m $\times$ 10 m as illustrated in Fig. 3. Table 3 provides the structural characteristics of the barge platform and the four identical OWCs.

**Table 3:** Standard barge and the four OWCs-based barge platforms’ features.

Parameter	Value
Platforms’ size (W L H)	40 m 40 m 10 m
Each OWC’s size (W L H)	5 m 5 m 10 m
Draft, Free board for both platforms	4 m, 6 m
Water displacement for the simple barge	6400 m$^3$
Water displacement for the barge with OWCs	6000 m$^3$
Mass, Including Ballast	5,452,000 kg
CM Location below SWL	0.281768 m
Roll Inertia about CM	726,900,000 kg$\cdot$m$^2$
Pitch Inertia about CM	726,900,000 kg$\cdot$m$^2$
Yaw Inertia about CM	1,453,900,000 kg$\cdot$m$^2$
Anchor (Water) Depth	150 m
Separation between Opposing Anchors	773.8 m
Unstretched Line Length	473.3 m
Neutral Line Length Resting on Seabed	250 m
Line Diameter	0.0809 m
Line Mass Density	130.4 kg/m
Line Extensional Stiffness	589,000,000 N

### Advanced hydrostatic and hydrodynamic computations

After designing the geometry of the newly proposed four OWCs-based barge platform, it is necessary to obtain the hydrodynamic and hydrostatic parameters. Therefore, the WAMIT numerical tool has been used to perform the advanced computations of these features. WAMIT is a diffraction panel program developed for the linear analysis of the interaction of surface waves with various types of floating and submerged structures. Hydrostatic and hydrodynamic coefficients have been obtained using the MultiSurf file directly into WAMIT to get the matrices $A_{Hydro}(\omega )$, $B_{Hydro}(\omega )$, $S_{Hydro}$ and $f_{Hydro}(\omega )$ as described in section “Theoretical background”. WAMIT calculates the hydrodynamic loads due to the water pressure on the wetted surfaces and can be linked to MultiSurf to use the geometry of the four OWCs-based barge model. Based on Gauss’ divergence theorem all hydrostatic data may be represented as surface integrals over the mean wetted body surface $S_b$. The following equation can describe the relationship between added mass and damping coefficients^61^:

32 $$\begin{aligned} A_{ij} - \frac{i}{\omega }B_{ij} = \rho \iint _{S_b} {n_i \varphi _j dS} \end{aligned}$$

The normalized added mass and damping coefficients can be calculated as:

33 $$\begin{aligned} \bar{A}_{ij}= & {} {{A_{ij} }}/{{\rho L^k }} \end{aligned}$$

34 $$\begin{aligned} \bar{B}_{ij}= & {} {{B_{ij} }}/{{\rho L^k \omega }} \end{aligned}$$

where *L* is the length scale and

35 $$\begin{aligned} {\left\{ \begin{array}{ll} k=3~~for &{}(i,j=1,2,3) \\ k=4~~for &{}(i=1,2,3, ~ j=4,5,6)~~or~~(i=4,5,6, ~ j=1,2,3) \\ k=5~~for &{}(i,j= 4,5,6) \end{array}\right. } \end{aligned}$$

The volume is defined as:

36 $$\begin{aligned} \forall = - \iint _{S_b } {n_1 x\;dS} = - \iint _{S_b } {n_2 y\;dS} = - \iint _{S_b } {n_3 z\;dS} \end{aligned}$$

The following are the coordinates of the center of buoyancy:

37 $$\begin{aligned}{}&x_b = \frac{{ - 1}}{{2\forall }}\iint _{S_b } {n_1 x^2 \;dS} \end{aligned}$$

38 $$\begin{aligned}{}&y_b = \frac{{ - 1}}{{2\forall }}\iint _{S_b } {n_2 y^2 \;dS} \end{aligned}$$

39 $$\begin{aligned}{}&z_b = \frac{{ - 1}}{{2\forall }}\iint _{S_b } {n_3 z^2 \;dS} \end{aligned}$$

The matrix of hydrostatic and gravitational restoring coefficients defined as:

40 $$\begin{aligned} S_{Hydro} = \left[ {\begin{array}{*{20}l} 0 &{} 0 &{} 0 &{} 0 &{} 0 &{} 0 \\ 0 &{} 0 &{} 0 &{} 0 &{} 0 &{} 0 \\ 0 &{} 0 &{} x_1&{} x_2 &{} x_3 &{} 0 \\ 0 &{} 0 &{} 0 &{}x_4 &{} x_5 &{} x_6 \\ 0 &{} 0 &{} 0 &{} 0 &{} x_7 &{} x_8 \\ 0 &{} 0 &{} 0 &{} 0 &{} 0 &{} 0 \\ \end{array} } \right] \end{aligned}$$

where $x_g, y_g, z_g$ are the coordinates of the center of gravity and $x_1$ to $x_8$ are:

$$x_1$$= $${\rho g\iint _{S_b }}$$,

$$x_2$$= $${\rho g\iint _{S_b } {yn_3 \;dS}}$$,

$$x_3$$= $${\rho g\iint _{S_b } {y^2 n_3 \;dS} + \rho g\forall z_b - mgz_g }$$,

$$x_4$$= $${-\rho g\iint _{S_b } {xyn_3 \;dS}}$$,

$$x_5$$= $${\rho g\forall x_b + mgx_g }$$

$$x_6$$=$${\rho g\forall x_b + mgx_g }$$

$$x_7$$= $${\rho g\iint _{S_b } {x^2 n_3 \;dS}}$$ and

$$x_8$$= $$- \rho g\forall y_b + mgy_g$$.

Two different barge platforms have been designed, one of them with open OWC and the other with closed OWC. Then, hydrodynamics (HydroDyn) and damping coefficients have been obtained for each platform using WAMIT, so that the resulting systems may be modelled within FAST. However, regarding WAMIT multiple bodies (NBodyMod = 2 or 3) calculations, WAMIT includes the capability to analyze multiple bodies that interact hydrodynamically and mechanically, which allows the use of separate WAMIT solutions for each body. Each of the separate bodies may oscillate independently with up to six degrees of freedom. The multiple WAMIT bodies can represent HydroDyn or the substructure flexibly using SubDyn. These bodies maybe coupled to FAST using SubDyn, a potential-flow solution, or HydroDyn, a finite-element beam model. In particular, for the purpose of this study case, two separate Geometric Data Files (GDFs) are needed^62^.

**Figure 4:**
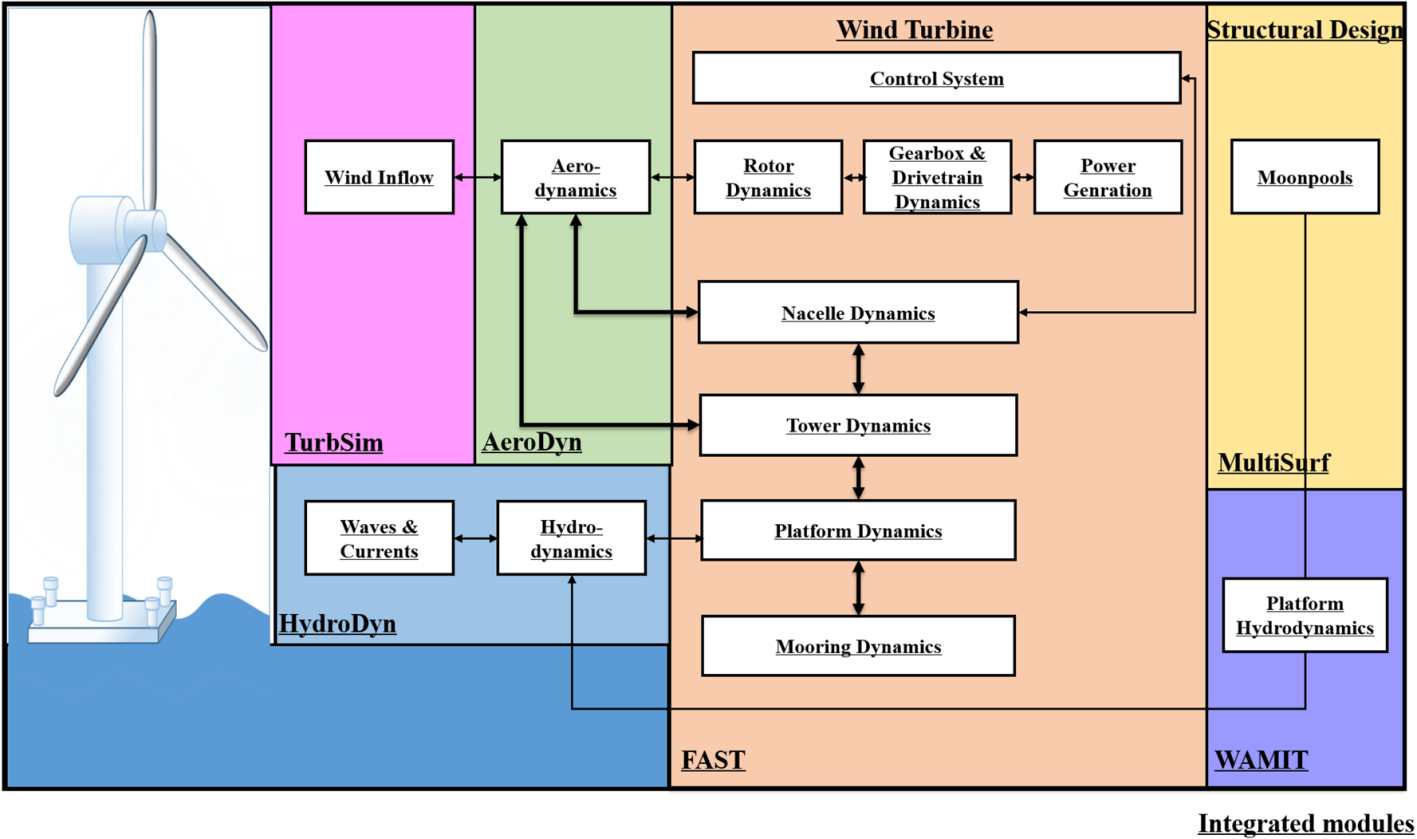
Interfacing modules to achieve aero-hydro-servo-elastic simulations.

The hydrodynamic data and added mass are obtained from WAMIT to be integrated into FAST. The procedure of interfacing modules to achieve aero-hydro-servo-elastic simulation has been described above in Fig. 4. The mooring lines’ tension depends on the buoyancy of the platform at hand, the cable weight, its elasticity, viscous-separation effects and the geometrical layout of the mooring system. In this sense, the aforementioned FAST software configures the catenary mooring lines system based on a quasi-static model, so that a time-domain model is used to compute the mooring tensions for a barge platform.

**Figure 5:**
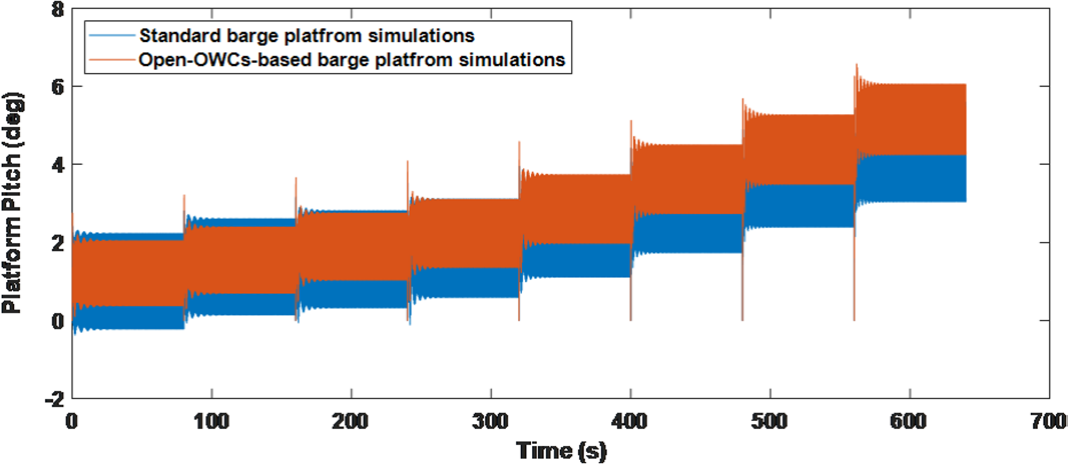
Standard based Barge Platform and 4-OWCs-based Barge Platform simulations.

Figure 5, which shows how oscillations are damped by deploying 4 OWCs in the barge platform. Therefore, the use of oscillating water columns as an active structural controller for the floating platform stabilization.

## ANN-based FOWT model

ANN is used to learn from data in order to make future predictions and is capable of recognizing patterns and making judgments based on previously stored information^63,64^. In this case, the row vectors of the inputs and weights are, *x*= $[x_1,x_2,\ldots ,x_n]$ and *w*=$[w_1,w_2,\ldots ,w_n]$ respectively. The bias $b_j$, which is a parameter in the ANN used to regulate the output, represents the deviations with respect to the mean value. The data has been transferred from the input layer to the output layer by the feed-forward network.

This transformation from the input to the output is defined with the activation function $\sigma _j$ as:

41 $$\begin{aligned} v_j=\sigma _j(s_j)=\sigma _j\left\{ \sum _{j=1}^{N}{w_{jk}x_k+b_j}\right\} \end{aligned}$$

where $s_j$ is a weighted sum of the inputs, establishing the connection between the $j\mathrm{th}$ neuron in a given layer, and the $k\mathrm{th}$ neurons in the previous layer, $b_j$ is the bias, and *N* is the total number of neurons.

**Figure 6:**
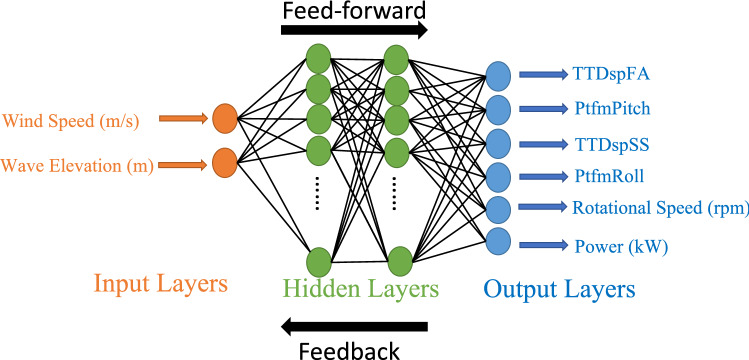
Proposed MLP network for the four OWCs-based barge FOWT system.

Several activation functions have been tested to train these networks according to the dynamics of the data. Finally, the ReLU activation function, which is defined as $max(0,s_j)$ has been used in the input layer, and for the hidden layers, the hyperbolic tangent activation function has been used, which is defined by $(e^{s_j}\text {-}e^{\text {-}s_j})/(e^{s_j}+e^{\text {-}s_j}).$ It is well established that single hidden layer networks, known as Vanilla Neural Networks^65^, perform poorly with highly non-linear and complex problems. On the other hand, network structures with more than one hidden layer, known as deep neural networks, offer better performance^66^. Therefore, due to the complexity of the proposed four OWCs-based barge FOWT system Multi-Layer Perceptron (MLP) has been employed as shown in Fig. 6.

Mean Squared Error (MSE) has been calculated to choose the best for our proposed FOWT model.

42 $$\begin{aligned} MSE=\frac{1}{n}\sum _{k}^{n}{(v_k\text {-}v_k^\prime })^2 \end{aligned}$$

where *n* is the total number of observations, $v_k$ is target output, and $v_k^\prime$ the estimated output by ANN.

For best results, the Levenberg-Marquardt algorithm (LMA)^24^ is used.

where the Jacobian matrix defined in Eq. (43) contains the first derivatives of the network errors with respect to weights and biases.

43 $$\begin{aligned} J(W) = \begin{pmatrix} \frac{\partial e_1(W)}{\partial W_1} &{}\frac{\partial e_1(W)}{\partial W_2} &{} \ldots &{} \frac{\partial e_1(W)}{\partial W_n}\\ \frac{\partial e_2(W)}{\partial W_1} &{} \frac{\partial e_2(W)}{\partial W_2} &{} \ldots &{} \frac{\partial e_2(W)}{\partial W_n} \\ \vdots &{} \vdots &{} \ddots &{} \vdots \\ \frac{\partial e_n(W)}{\partial W_1} &{} \frac{\partial e_n(W)}{\partial W_2} &{}\ldots &{} \frac{\partial e_n(W)}{\partial W_n} \\ \end{pmatrix} \end{aligned}$$

where *e* is the network errors and *W* is the weights vector.

The gradient of performance in our case may be defined as:

44 $$\begin{aligned} \nabla f(W)=2J^T(W).e(W) \end{aligned}$$

The LMA is used here to take into account both curvature and slope for faster convergence. Thus, it performs the modified gradient descent :

45 $$\begin{aligned} W_{k+1}=W_k-\left[ J^TJ+\mu I \right] ^{-1}J^Te \end{aligned}$$

where $\mu$ is the learning rate and *I* is the identity matrix.

**Figure 7:**
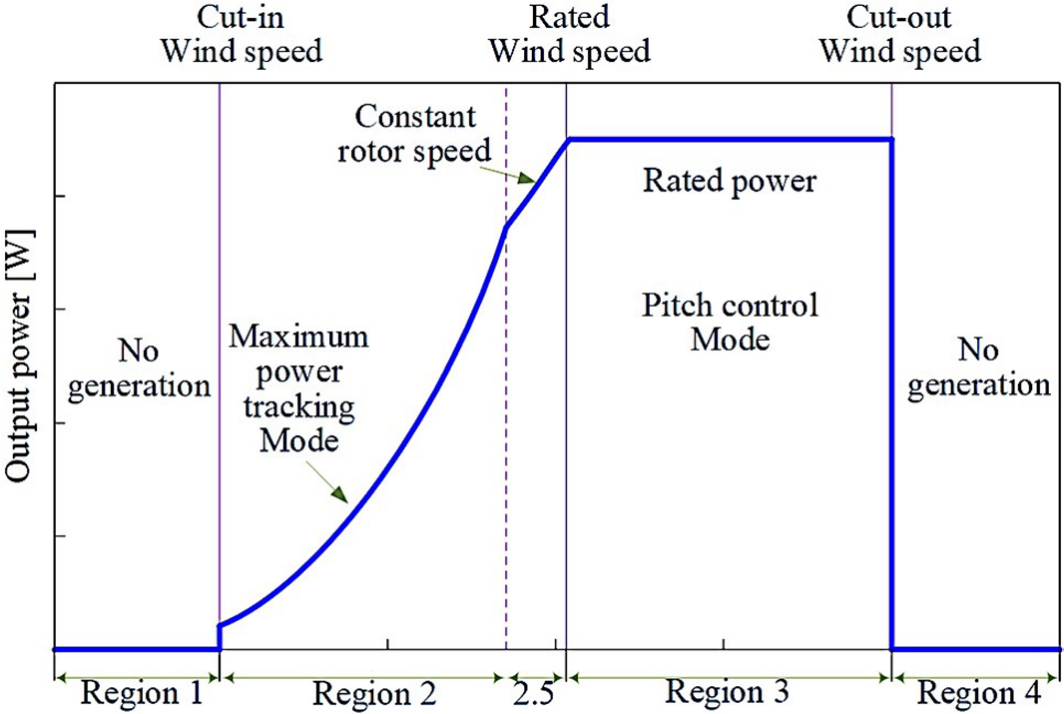
Operating regions in wind turbines.

**Figure 8:**
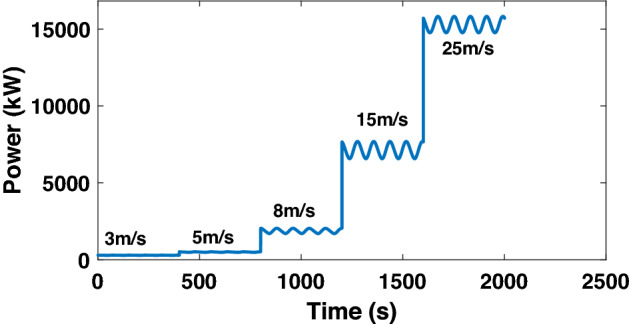
Full operating range response test for the uncontrolled model.

There are four operating regions for wind turbine operation^67^, (see Fig. 7). In Region 2, corresponding usually to MPPT mode, the generator and rotor speeds increase with wind speed to maintain a constant tip-speed ratio and optimal wind-power conversion efficiency, while in Region 3 the wind turbine operates at rated power due to a strong power limitation pitch control action. On the other hand, there is no power generation in regions 1 and 4 as may be observed in see Fig. 7. In this context, please note that the aim of the proposed ANN scheme is to capture and match the dynamical behavior of the structure. In this sense, since the control strategies will limit the power to 5MW, there was no particular interest in including simulations in the full operating region (3-25m/s) as shown in Fig. 8. Therefore, the data sets employed are those of the regions presenting the richest dynamical diversity. That is, comprising 8-15m/s wind speed representative ranges within those regions. Meanwhile, the control of the pitch angle has been removed to model the real behavior of the system in the absence of a controller.

Furthermore, as indicated previously, the main objective of the work is to use ML algorithms to replicate the complex hybrid dynamics of the FOWT-OWCs combined structure, allowing the use of feedback controls. However, it would be an excellent idea in the future to extend the use of ML approaches to study other aspects such as such as damping from the oscillating water columns.

## Computations and results

**Figure 9:**
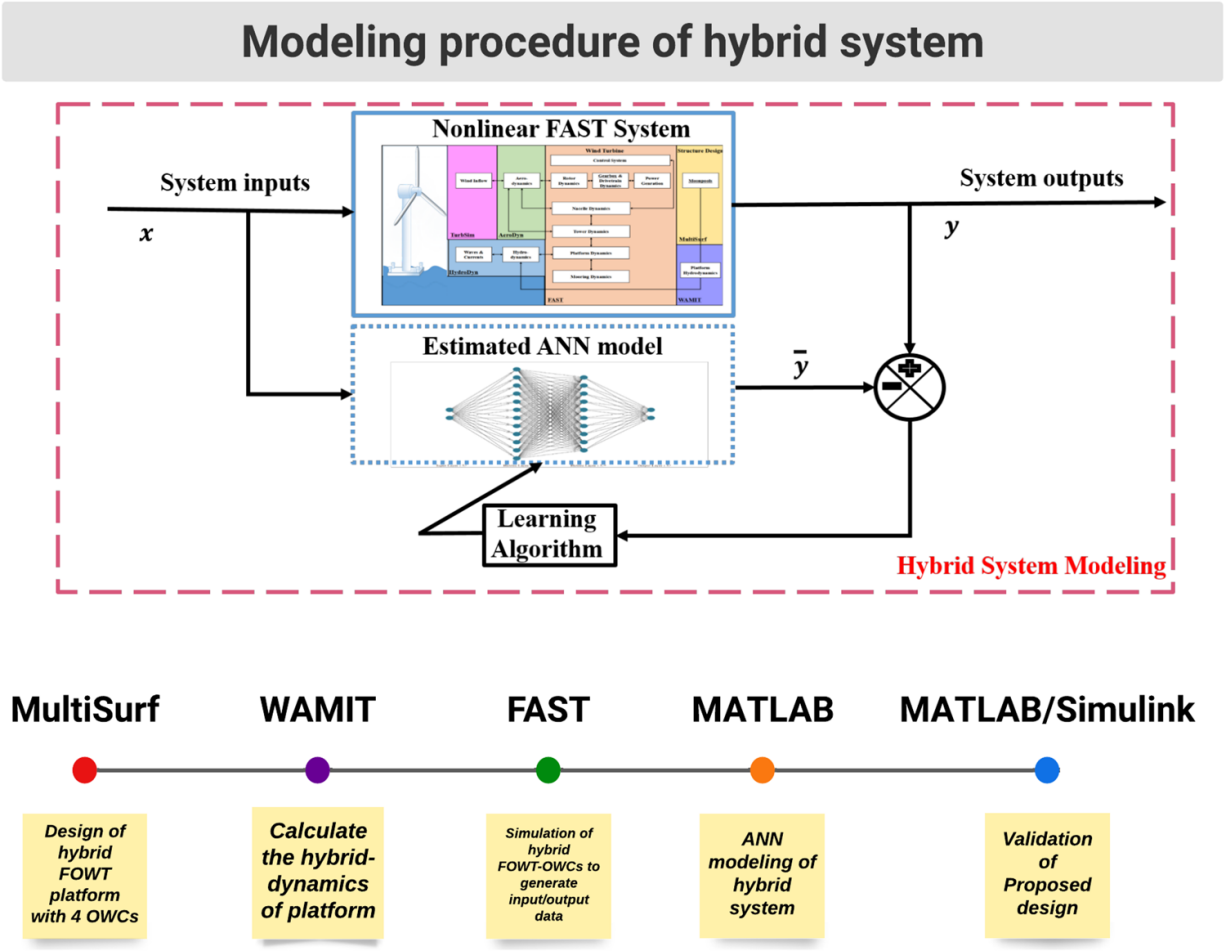
Modeling procedure of the hybrid system.

The structure for collecting, simulating and developing the network has been described in the diagram of Fig. 9. MultiSurf is used to define the platform’s structural geometry. Then, the hydrodynamic forces, added mass, damping coefficient, and hydrostatic matrices are then calculated using WAMIT. These matrices are introduced to FAST to compute the aerodynamic characteristics and behaviour of the 5MW floating offshore wind turbine. In particular, simulations for wind speeds from 8 to 15 m/s provide different dynamic responses of the FOWT structure.

The data set of two inputs, wind speed and wave elevation has been considered to train the ANN. These models replicate the structural behavior of a FOWT and its power dynamics with an output vector defined as:

46 $$\begin{aligned} v_k^{\prime ~T}= \left[ {Pitch} ~~{Fore\text {-}aft} ~~{Roll} ~~{Side\text {-}to\text {-}Side} ~~{Rotational}-{speed} ~~{Power}\right] \end{aligned}$$

The 6 DOF dynamics: translational modes are; tower fore-aft and side-to-side displacements, rotational mode; platform roll and platform pitch angles, generated power and generator rotational speed are considered by enabling the aero-hydrodo-servo-dynamics. The demanding tasks of dynamical matching have been performed by training the ANN model. The network comprises two inputs and six outputs (2 $\times$ 6), whereas the wave elevations and wind speeds are inputs to the network as shown in Fig. 13a and b. The estimated outputs are platform pitch, platform roll, side-to-side and fore-aft displacements, generator rotational speed, and the generated power. The prediction of heave motion using the proposed ANN model has not been addressed. Although heave motion is quite relevant in stand-alone off-shore OWC-based PTOs where a resonant condition is even desired for MPPT maximum power production, the objective of this work does not relay in such a strong way in this aspect, so that its particular study^68^ has not been stressed.

### Training results

In order to achieve the best performance, simulations were carried out to find the best fit with the help of a multi-layered perceptron. The data have been divided into three parts: 70% of all data for training, 15% for validation, and 15% for testing. Muti-layered networks are capable of performing just about any linear and nonlinear computations and can approximate any reasonable functions arbitrarily well. Such networks overcome the problems associated with perceptions and linear networks.

**Figure 10:**
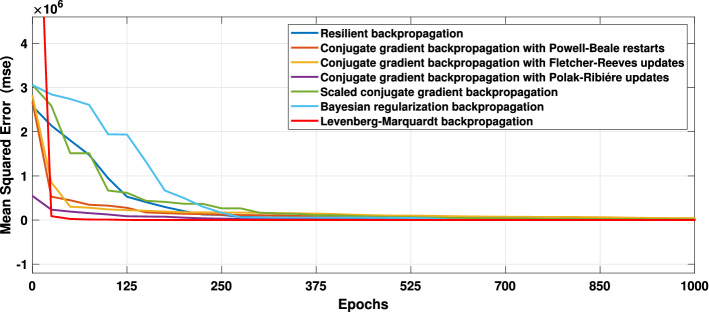
Errors graphs for validation.

Several training functions are examined to train the network, for example, **trainlm**, is frequently the fastest backpropagation method and is strongly recommended as a first-choice supervised technique, despite requiring more memory than other algorithms. A **trainrp** is a network training function that uses the robust backpropagation (Rprop) algorithm to update weight and bias variables. The resilient backpropagation training technique tries to eliminate the negative impacts of partial derivative magnitudes. All conjugate gradient algorithms attempt the steepest descent direction on the first iteration (negative of the gradient). A **traincgb** is a network training function that uses conjugate gradient backpropagation with Powell-Beale restarts^69^ to update weight and bias values. The search direction is regularly reset to the gradient’s negative in all conjugate gradient algorithms. **trainbr** is a network training function that uses Levenberg-Marquardt optimization to update the weight and bias variables^70^. It minimizes a combination of squared errors and consequences before determining the best combination to create a generalizable network. The procedure is known as Bayesian regularization. The best results are found activating Levenberg-Marquardt^24^ optimization techniques as shown in Fig. 10. The ANN models have 5 hidden layers in which the neurons in the hidden layers use sigmoid as the activation function and this network uses linear activation function (ReLU) in the output layer. In order to get satisfactory results, the lowest MSE has been targeted.

**Figure 11:**
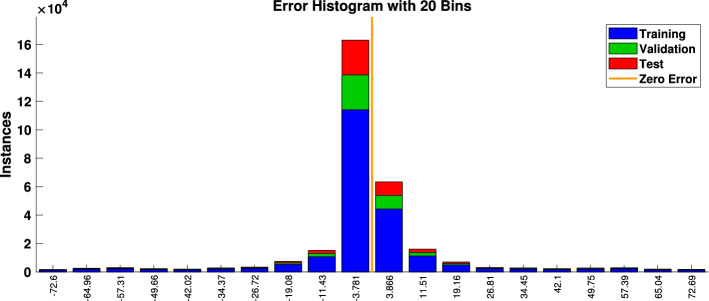
Errors histogram for validation with LMA training function.

**Figure 12:**
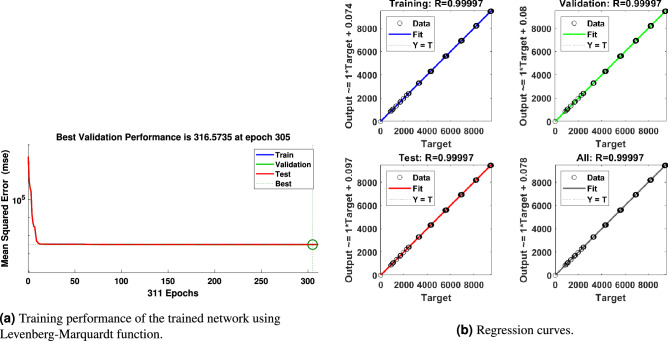
ANN inputs.

The results obtained after using various training functions are summarized in Table 4. The first column contains the network training function, the second column is for performance, followed by MSE values, and the number of epochs. The plot of the trained feed-forward network error values histogram is shown in ﻿Fig. 11. It illustrates the distribution of the degree of errors. Most of the errors are closest to zero, with very few deviations from that. The graphs for training, testing, and validation for ANN are shown in Fig. 12a. It can be seen that the best validation performance has been found for MSE is equal to 316 at 305 epochs employing the LMA training function. The regression curves are also close to one, which implies that the estimated ANN data mimic perfectly the target data as illustrated in Fig. 12b. The black lines for training, green curves for validation, and red curves for testing have been labeled in these figures.

**Table 4:** History of training performance employing varied training functions.

Training function	Performance	MSE	R	Epochs	Training function	Performance	MSE	R	Epochs
trainrp	Validation	542	0.9994	479	trainscg	Validation	341	0.9998	914
	Training		0.9994			Training		0.9999	
	Test		0.9995			Test		0.9998	
traincgb	Training	428	0.9995	675	trainbr	Training	319	0.9997	942
	Validation		0.9996			Validation		0.9998	
	Test		0.9996			Test		0.9998	
traincgf	Training	351	0.9998	343	trainblm	Training	316	0.9999	953
	Validation		0.9996			Validation		0.9999	
	Test		0.9998			Test		0.9999	
traincgp	Training	344	0.9996	513					
	Validation		0.9997						
	Test		0.9996						

### Validation results

Once the ANN models have been developed and trained, they need to be adequately validated. Results, shown in Figs. 14, 15 and 16, demonstrate the effectiveness of the designed ANN models. The red curve (solid) corresponds to the ANN-based model and the black curve (dashed) represents the FAST model. For this type of system, a periodic steady-state condition was found by simulating the nonlinear model long enough to dampen out the transient state. Therefore the first 500s of simulations have been omitted to avoid transients.

**Figure 13:**
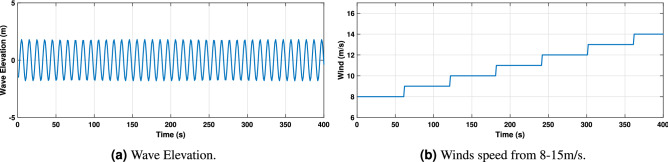
ANN inputs.

**Figure 14:**
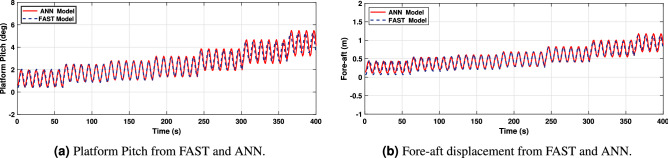
Platform‘ Pitch angle and top-tower displacement.

Figure 14a and b correspond to the platform pitch angle and fore-aft displacement. As it may be observed, they reveal that the model has been adequately trained and that there is a high agreement between the values obtained from FAST and the proposed ANN model, with slight differences for non-representative low wind speeds.

**Figure 15:**
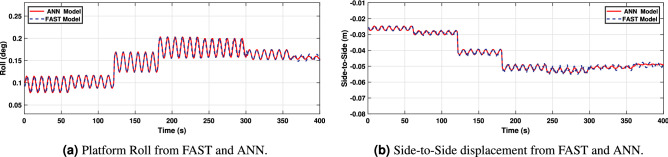
Platform‘ Roll angle and side-to-side displacement.

In turn, Fig. 15a and b display the platform roll and side-to-side displacement. It may be observed also how the ANN model outputs, successfully match the behavior of the FAST model but, with slight differences for some wind speeds in the platform roll and side-to-side displacement. Nevertheless, it should be noted that this composes a minor error due to the reduced movement range since the system inputs have a negligible effect on platform roll and fore-aft displacement.

**Figure 16:**
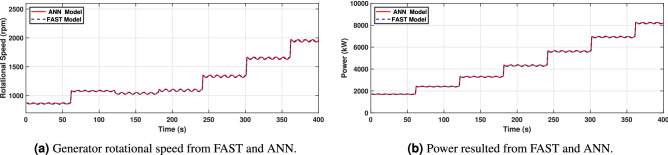
Generator rotational speed and power.

In Fig. 16a and b, the results show that as the wind speed increases, the generated rotational speed increases and the power exceeds its nominal value, which is 5MW. It can also be observed that, at the wind speed of 15m/s, the power increases to around 8MW due to the absence of pitch angle control, or torque control.

### Irregular wave scenario

In this subsection, a nonlinear irregular wave model appropriate for shallow water depths, where some offshore wind turbines are sited, has been developed in order to improve the accuracy of the coupled system simulations. To do so, the nonlinear irregular wave model is incorporated into the coupled aero-servo-hydro-elastic simulation of a hybrid FOWT-OWCs system. The FAST numerical tool is re-simulated under irregular wave conditions for wind speeds of 8–15 m/s to study the dynamic behavior of the structure. The ANN1 model is then developed and trained for these irregular wave conditions.

**Figure 17:**
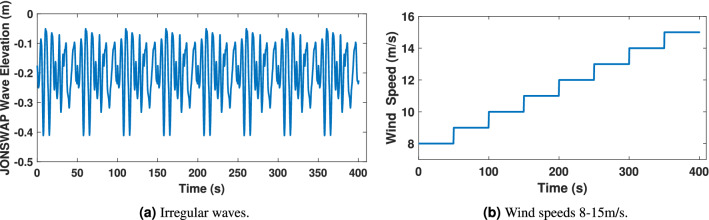
ANN input.

**Figure 18:**
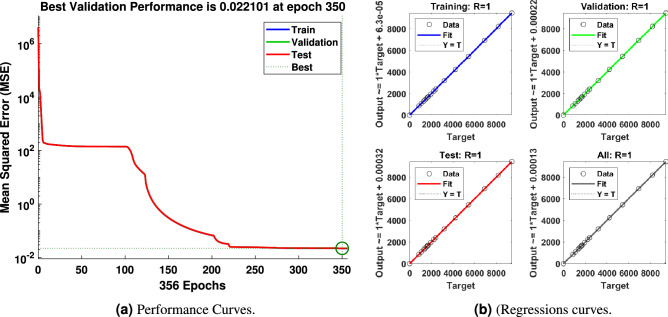
Tanning performance and regressions curves.

**Figure 19:**
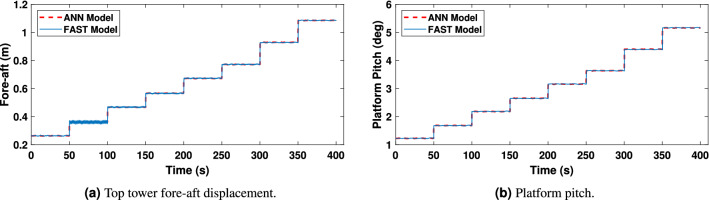
Top tower fore-aft displacement and platform pitch in irregular wave conditions.

**Figure 20:**
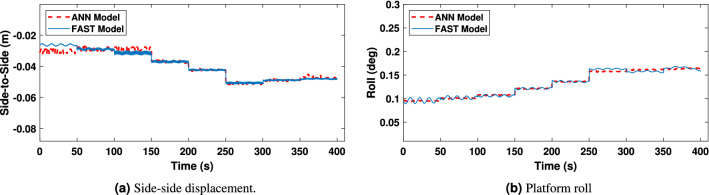
Side-to-side displacement and platform roll in irregular wave condition.

**Figure 21:**
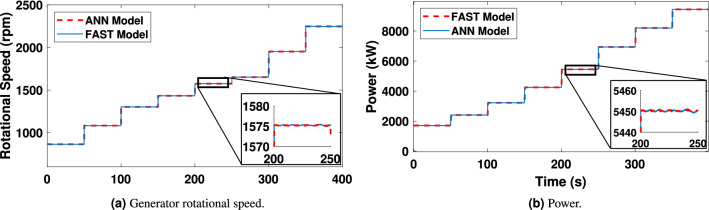
Generator rotational speed and power in irregular wave conditions.

The network consists of two inputs and six outputs. The network’s inputs are shown in Fig. 17, and its estimated outputs are shown in Figs. 19, 20 and 21. ANN1 model comprises 5 hidden layers in which there are neurons hidden layers that use sigmoid as an activation function and this network uses linear activation function (ReLU) in the output layer. For good results, the lowest MSE has been targeted. In Fig. 18a, it can be seen that the best validation performance has been found for MSE equal to $2.241 \times 10^{- 2}$ at 350 epochs employing the LMA training function. The regression curves obtained R = 1, which demonstrates that the network has been trained adequately.

As it can be observed from Figs. 19, 20 and 21, the model has been properly trained and validated, showing a strong correlation between the results from FAST and the suggested ANN1 model under irregular wave conditions. In particular, side-to-side displacement and platform roll angle are shown in Fig. 20a and b, respectively, with minor variations for low-high wind speeds.

In all of these figures, it is clearly demonstrated that the proposed ANN model presents an excellent agreement with the gold standard software FAST for various conditions and functions flawlessly in tough conditions. Therefore, as it has been proven, the hybrid platform ANN composes a reliable model that enables closed-loop control implementation and, in particular, for platform stabilization feedback control.

## Conclusion

This work presents the design and validation of an artificial neural network-based model of a hybrid floating offshore wind turbine with integrated oscillating water columns. The data has been obtained by incorporating FAST hydrodynamics, aerodynamics, and servo-dynamics properties for the whole hybrid system. The aim of the proposed ANN model is to match the behavior and structural performance of the hybrid FOWT-OWCs system. To achieve this objective, the model has been trained with adequate parameters taking into account the lowest MSE so as to enable closed-loop control of the hybrid FOWT system. The model has been then benchmarked for diverse wind speed and wave scenarios in order to demonstrate its computational efficiency, validity, and accuracy, comparing the obtained ANN-based FOWT model’s outputs to those of the complete non-linear complex FAST model.

The results show that the proposed control-oriented ANN model is highly accurate at predicting the power, tower displacements and its translational and rotational modes. In this way, the forecasting and predictive capabilities of the ANN model compose an efficient and promising alternative to model complex systems, as is the case of FOWT-OWCs hybrid systems, facilitating future investigations for the implementation of platform stabilization closed-loop control. The proposed methodology can be useful, as a supportive tool, in the studies of offshore wind farm designers.

This work will be extended in the future to incorporate advanced machine learning control algorithms with a feedback loop to reduce unwanted platform motions. In addition, this work will be expanded to include uncertainties and irregular waves for robust control designs of undesired platform vibration modes control of hybrid systems.

## Data Availability

All data generated or analyzed during this study are included in this published article and its supplementary information files.
